# Subchronic toxicity of Nile tilapia with different exposure routes to *Microcystis aeruginosa*: Histopathology, liver functions, and oxidative stress biomarkers

**DOI:** 10.14202/vetworld.2017.955-963

**Published:** 2017-08-21

**Authors:** H. M. R. Abdel-Latif, A. M. Abou Khashaba

**Affiliations:** 1 Department of Poultry and Fish Diseases, Faculty of Veterinary Medicine, Alexandria University (Matrouh Branch), Fuka City, Box: 51744, Matrouh Province, Egypt; 2 Department of Food Inspection, Animal Health Research Institute, Dokki, Giza Province, Egypt

**Keywords:** catalase, lipid peroxidation, *Microcystis aeruginosa*, microcystins, Nile tilapia

## Abstract

**Background:**

Toxic cyanobacterial blooms (*Microcystis aeruginosa* contains microcystins [MCs]) have been reported to induce clinicopathological alterations as well as different oxidative stress in aquatic biota.

**Aim:**

Three-week subchronic exposure experiment was carried out on Nile tilapia, to determine their effects on fish behavior, tissues, liver functions, antioxidant enzymes, and lipid peroxidation.

**Materials and Methods:**

Fish were exposed to four main treatments; orally fed diet plus toxic cells of *M. aeruginosa* (containing 3500 µg/g MC-LR), immersion in 500 µg MC-LR/L, intraperitoneal injection of *M. aeruginosa* MC-LR with a dose of 0.1 ml of extracted toxin at a dose of 200 μg/kg bwt, and the fourth one served as a control group, then the fish were sacrificed at the end of 3^rd^ week of exposure.

**Results:**

The results revealed no recorded mortality with obvious behavioral changes and an enlarged liver with the congested gall bladder. Histopathology demonstrated fragmentation, hyalinization, and necrosis of the subcutaneous musculature marked fatty degeneration, and vacuolation of hepatopancreatic cells with adhesion of the secondary gill lamellae associated with severe leukocytic infiltration. Furthermore, liver functions enzymes (aspartate aminotransferase and alanine aminotransferase, and the activities of glutathione peroxidase, glutathione reductase, lipid peroxidase, and catalase enzymes) were significantly increased in all treatments starting from the 2^nd^ week as compared to the control levels.

**Conclusion:**

In this context, the study addresses the possible toxicological impacts of toxic *M. aeruginosa* contain MC-LR to Nile tilapia, and the results investigated that MC-LR is toxic to Nile tilapia in different routes of exposure as well as different doses.

## Introduction

Nile tilapia is one of the major cultured species worldwide, particularly in Egypt [1], with a yearly growth rate of about 12.2% [2]. Toxic cyanobacterial algal blooms (especially *Microcystis aeruginosa*) produce potent cyanotoxins known as microcystins (MCs) [3,4], which could be accumulated in fish tissues and organs from freshwater lakes [5,6]. MCs-LR is the predominantly potent hepatotoxin for aquatic biota [7,8] and possibly cause mass kills of fish [9].

Several reviews were demonstrated the toxicity of these toxic cyanobacterial blooms containing MC-LR on Nile tilapia in various localities in Egypt, whereas heavy kills were at Sharkia, Alexandria, the Nile River, and several irrigation canals [10-13], and there are yearly greater records of these blooms; this may be due to the high water temperature throughout the year and the abundance of environmental pollutants.

The toxicological impacts of the cyanobacteria toxic algae and their toxins in fish were widely assessed and discussed before, whereas numerous hepatotoxicity and renal toxicity histopathological signs were demonstrated in *Cyprinus carpio* [14], *Oreochromis mossambicus* [15], and in *Oreochromis niloticus* [16]. In addition, one of the biochemical characteristics of MCs toxicity is the production of reactive oxygen species, which is responsible for oxidative stress responses of the exposed fish [17].

The antioxidant enzymes were elevated with the MC-LR toxicity in Nile tilapia [18-20] and in early life stages of the zebrafish [21]. The previous reviews were based mainly on time-dependent manner. Therefore, this study was to demonstrate the impacts of subchronic exposure of these toxic cyanobacteria and their MC (MC-LR) in terms of histopathological findings, lipid peroxidation (LPO), and oxidative stress biomarkers of Nile tilapia based on time- and dose-dependent manners using different three main experimentally exposure toxicity (immersion, feed incorporation, and intraperitoneal injection).

## Materials and Methods

### Ethical approval

The approval from the Institutional Animal Ethics Committee to carry out this study was not required as no invasive technique was used.

### Experimental design and fish rearing

This study was conducted on 160 fish, Nile tilapia (*O. niloticus*) with mean weight 50±10 g. They were obtained from a private farm in Kafr El-Sheikh Province, Egypt, and were transferred as soon as possible to the laboratory where they were held in eight aquaria, each with 60 L of fresh chlorine-free tap water (overnight 3 days prior the experiments). The aquaria were set up with continuous water siphoning and pump aerators, and the temperature was kept constant (24±3°C). Dissolved oxygen values were maintained in between 4.5 and 5.0 mg/L. Fish were left to be acclimated 2 weeks prior the start of the experiments. Fish were hand fed on a commercial diet containing 28% protein 2 times daily, 3% of body weight of fish, and fish wastes were siphoned weekly.

### Toxic algae

#### The assemblage of algal cells and MCs

MCs were extracted from dried *M. aeruginosa* algal blooms, which thereby collected from the drainage canal of Mariout Lake, Egypt. Lyophilized algal cells (50 mg) were extracted 3 times with 10 ml of 0.1 M acetic acid, and 20 ml of a combined mix of both chloroform and methanol added (1:1 v/v). The algal suspension was sonicated in an ultrasound bath for 15 min, stirred for 30 min at room temperature, and then centrifuged at 4500 rpm for 15 min.

#### Demonstration of MCs: Types and quantity

High-performance liquid chromatography analysis was used for separation of the crude extract concentrations of MCs, using standard solutions of MCs (Novabiochem, Nottingham, UK) prepared in methanol (500 µg/ml) and diluted as required with methanol for use as working solutions (0.5-5.0 mg/L of each toxin) [22], whereas the main toxin of MCs produced is (MC-LR). MC-YR and MC-LF were not found in our isolated strain from the examined locality. Furthermore, MC-RR was found in small quantity (<0.50% of the total toxins produced/g dry algae).

### Toxicity bioassay

#### Subchronic in vivo exposure and experimental designs

Fish were experimentally exposed to cyanobacterial toxic algal cells containing MC-LR for 3 weeks’ *in vivo* exposure period by different exposure methods; intraperitoneal injection of MC-LR (IPM), immersion bath in MC-LR (IMM), and incorporation of dried cyanobacterial cells in fish diet. Fish were divided (20 individuals/aquarium). Each exposure treatment has two replicates (one exposure and the other is control [CTR]).

### Experiment I (IPM)

Fish were intraperitoneally (IP) injected with 0.1 ml of extracted MC-LR at a sublethal dose of 200 µg/kg [15,23,24], while the control group was IP injected with sterile 0.9% saline solution.

### Experiment II (IMM)

Fish were immersed in the crude extract from *M. aeruginosa* containing MC-LR equivalent 500 µg/L, while the control group was left in the aquarium water without MC-LR toxins [21].

### Experiment III (feeding toxic cyanobacterial cells in pelleted diets [FC])

Lyophilized cyanobacterial cells (containing 3500 µg/g MC-LR) were incorporated in fish diets, whereas the toxic algae were manually crushed in a mortar followed by sonication. Fish were fed with a dose rate of 60 µg MC-LR/fish/day, for 21 days [18].

### Clinical examination and sampling of the exposed fish

During the three experiments, fish were observed for any clinical signs and behavioral abnormalities. After the end of 3^rd^ week of exposure, fish were sacrificed for a demonstration of any postmortem (PM) lesions.

After the exposure period, fish were sacrificed and sampled for histopathological examination. In addition, serum and specimens from gills, liver, kidneys, and muscular tissues were collected from different groups for the determination of LPO, liver function enzymes, and oxidative stress biomarkers.

### Kits for biochemical analysis

Kits for liver function tests (alanine aminotransferase [ALT] and aspartate aminotransferase [AST]), kits for lipid peroxide (malondialdehyde [MDA]), and for oxidative stress biomarkers are glutathione peroxidase (GPx), reduced glutathione, and catalase (CAT) were purchased from Biodiagnostic and Biotechnology Co., Egypt.

### Bleeding and serum collection

Blood samples were collected weekly for 3 successive weeks from the caudal blood vessels. Fish body surface was wiped and dried out. Samples (3 ml/fish) were assembled, relocated into Eppendorf tubes for serum collection [25], whereas serum detached by centrifugation at 2000 rpm for 10 min and then stored at −20°C till being used [26] for spectrophotometric measuring of enzymes of liver functions as AST and ALT (Lab. American Inc., USA).

### LPO and oxidative stress biomarkers

#### Preparation of post-mitochondrial supernatant (PMS)

At the end of the experiments, the fish were sacrificed, and the liver, kidney, and gills were removed, weighed, rinsed with ice-cold physiological saline (0.9% NaCl), (the gill filaments were trimmed from the gill arches, and the arches were discarded). The tissues were homogenized in chilled Tris buffer (100 mM, pH 7.8; 1:10 w/v) using tissue homogenizer. The homogenates were centrifuged at 1000 g for 30 min at 4°C to obtain the PMS for various biochemical analyses.

#### LPO

It was determined by assessing the MDA concentration (the index of LPO) using the thiobarbituric acid (TBA) color reaction [27], this mixture was then heated at a temperature of 95°C for 30 min to form a TBA-reactive product. The absorbance of the resultant pink product was considered at 532 nm. The values were determined as nmol MDA/mg protein.

### Antioxidant enzymes

#### CAT enzyme activity

The CAT enzyme was assayed [28]. It reacts with a known quantity of H_2_O_2_, and the reaction is stopped after 1 min with a CAT inhibitor. In the presence of peroxidase, the remaining H_2_O_2_ reacts with 3,5-dichloro-2-hydroxybenzenesulfonic acid and 4-aminophenazone to form a chromophore, with a color intensity inversely proportional to the amount of CAT in the sample. The absorbance was measured spectrophotometrically at 510 nm. Results are expressed in terms of nmol H_2_O_2_ consumed/min/mg protein.

#### GPx and glutathione reductase (GR) activities

The GPx activity was measured spectrophotometrically and expressed as unit per milligram of protein (U/mg prot). One unit of GPx represents 1 µmol oxidized nicotinamide adenine dinucleotide phosphate (NADPH)/min [29].

GPx activity was assayed by following the rate of NADPH oxidation at 340 nm by the coupled reaction with GR. The specific activity was determined using the extinction coefficient of 6.22 mM^−1^ cm^−1^ [30].

GR activity was determined spectrophotometrically by measuring NADPH oxidation at 340 nm [31].

### Histopathological studies

Tissue samples (livers, gills, and musculature) were collected from fish of the experimental and control groups after the end of the exposure period (3 weeks) and then were rapidly fixed inadequate amount 10% neutral buffered formalin for several hours, dehydrated, paraffin-embedded, and archived. Paraffin blocks were prepared, and sections of 3-5 mm were mounted and stained with hematoxylin and eosin stains [32].

### Statistical analysis

Results are presented as the mean±standard error. The differences between the data of biochemical analysis from the different exposure experiments for 3 weeks were statistically analyzed using a t-test, Duncan-test after ANOVA and simple correlation [33] to examine the significant effect of different concentrations and route dependent of cyanobacterial cells containing MC-LR on the studied parameters.

## Results

### Mortality, clinical signs, and PM lesions

No fish died throughout the whole exposure period in the three experiments. The exposed fish have been behaved like the control one, except in some fish, whereas there were obvious changes in swimming, lethargy, accumulation to one side of the aquaria, and rest on the aquaria floor. Moreover, there were some external alterations observed in some fish as detached scales, slight ascites, slight exophthalmia, opaqueness, and pale skin coloration. Some fish showing erythematic or hemorrhagic patches around the mouth, operculum and pectoral fins, and skin ulcers penetrate to the underlying musculature.

Gross pathological changes observed were severely congested gills, swollen congested liver with hemorrhagic patches in tips, with the congested engorged gall bladder.

### Histopathological findings

**Figure-1:**
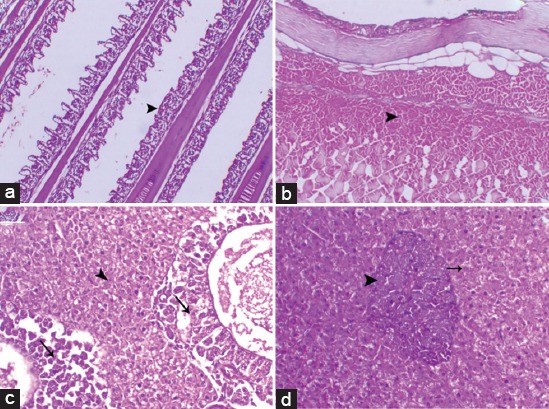
Photomicrographs of Group III (feeding cyanobacterial cells in the diet of Nile tilapia) showed marked adhesion of the secondary gill lamellae (arrowhead) with severe leukocytic infiltration (a), the subcutaneous layer with marked fragmentation of the underneath muscle fibers (arrowhead) with depletion of the goblet cells (b), and marked vacuolation and necrosis of the pancreatic portion (arrow), and the most of the hepatocytes were with vesicular nucleus with certain vacuolation mostly around the pancreatic portion (arrowhead) (c), with proliferated both pancreatic and hepatic cells (arrowhead and arrow, respectively) (d) (H and E stain, 200×).

**Figure-2:**
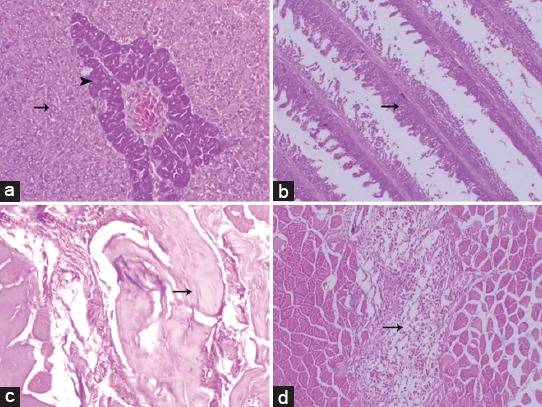
Photomicrographs of Group I (intraperitoneal injection of microcystin [MC-LR]) showed marked hyperplasia of pancreatic and hepatic cells (arrowhead and arrow, respectively) (a), adhesion of the gill lamellae (arrow) (b), and the subcutaneous layer of fish injected with MC at dose 500 µg/kg showing marked fragmentation of the muscle fiber(arrowhead), and hyalinization (arrow) (c), with showing focal large necrotic area within the muscle associated with marked leukocytic infiltration (arrow) (d), (H and E stain, 200×).

**Figure-3:**
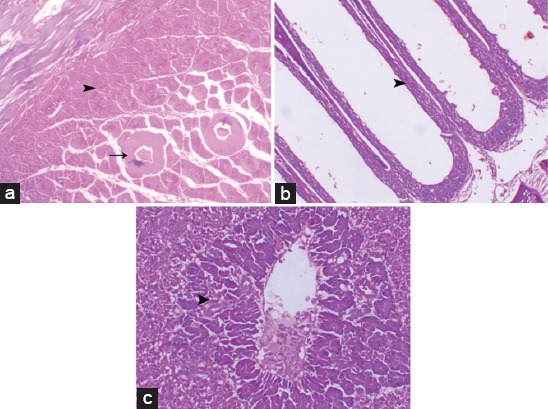
Photomicrographs of Group II (Nile tilapia fish treated with microcystin [MC-LR]) (immersion group) showed the subcutaneous layer of showing massive necrosis of subcutaneous muscle (arrow), marked fragmentation and vacuolation of the muscle fibers and interstitial edema (a), the gills showed complete adhesion of the secondary lamellae (arrowhead) (b), and hepatic degeneration (arrowhead) (c), (H and E stain, 200×).

The histopathological lesions of the exposed Nile tilapia (Figures-1-3) were, interestingly, varied by the dosage and route of administration.

### Effect of cyanobacteria cells on liver functions enzymes

Results in Tables-1 and 2 cleared that there is a significant difference (p<0.05) of the serum AST and serum ALT levels of *O. niloticus* regarding the exposure routes among the 3 weeks of exposure, whereas their levels were elevated in all exposed groups than that observed in control (CTR) group. In addition, their levels were elevated steadily from the 1st week to the 3^rd^ week of the experiment.

**Table-1:** Effects of cyanobacterial cells and MC-LR on serum AST (IU/L) of *O. niloticus* by different exposure routes.

Exposure period	Mean±SE			

	CTR	IPM	IMM	FC
1^st^ week	37.87±0.90^A^	53.65±1.22^C^	45.78±0.65^C^	41.90±0.89^C^
2^nd^ week	36.41±0.68^B^	60.87±1.23^B^	50.84±0.73^B^	42.26±0.37^B^
3^rd^week	36.15±0.29^C^	63.37±1.53^A^	54.80±0.93^A^	43.35±0.84^A^

Means within the same column of different letters are significantly different at p<0.05. *O. niloticus=Oreochromis niloticus*, IPM=Intraperitoneal injection of MC-LR, IMM=Immersion bath in MC-LR, FC=Feeding toxic cyanobacterial cells in pelleted diets, SE=Standard error, MC=Microcystins, AST=Aspartate aminotransferase

**Table-2:** Effects of cyanobacterial cells and MC-LR on serum ALT (IU/L) of *O. niloticus* by different exposure routes.

Exposure period	Mean±SE			

	CTR	IPM	IMM	FM
1^st^ week	40.42±0.47^C^	53.79±1.03^D^	49.63±0.28^C^	46.08±0.49^B^
2^nd^ week	43.03±0.61^B^	62.65±0.26^B^	54.63±0.54^B^	46.13±0.31^B^
3^rd^ week	45.52±0.64^A^	65.41±0.28^A^	57.23±0.74^A^	47.34±0.08^A^

Means within the same column of different letters are significantly different at p<0.05. *O. niloticus=Oreochromis niloticus*, IPM=Intraperitoneal injection of MC-LR, IMM=Immersion bath in MC-LR, FC=Feeding toxic cyanobacterial cells in pelleted diets, SE=Standard error, MC=Microcystins, ALT=Alanine aminotransferase

### Effect of cyanobacterial cells containing MC-LR on LPO and antioxidant enzymes

Results investigated that there is a significant difference (p<0.05) of the MDA levels (Table 3), and the activities of GR (Table 4), Gpx (Table 5), and CAT enzyme (Table 6), in the serum, kidney, and muscular tissue of the exposed fish regarding the exposure routes among the 3 weeks of exposure, whereas their levels elevated in all exposed groups than that observed in control (CTR) group. Furthermore, it was found that their levels in the serum, liver, and kidney samples were higher than that of gills and musculature. In addition, they were elevated steadily from the 1^st^ week to the 3^rd^ week of the experiment.

**Table-3:** Effects of cyanobacterial cells containing MC-LR on the MDA levels (nmol [µM MDA/mg protein]/g wet tissue) in the serum, gills, liver, kidney, and muscular tissue of *O. niloticus* by different exposure routes.

Exposure period	Sample	Mean±SE			

		CTR	IPM	IMM	FC
1^st^ week	Serum	26.78±0.63^A^	30.27±0.57^A^	28.70±0.34^A^	32.79±0.45^B^
	Gills	8.93±0.31^C^	16.29±0.63^C^	10.43±0.12^D^	8.66±0.27^D^
	Liver	11.57±0.26^C^	30.47±0.15^A^	26.00±0.42^A^	28.40±0.60^A^
	Kidney	18.42±0.63^B^	21.09±0.27^B^	19.32±0.37^C^	17.45±0.42^C^
	Musculature	27.53±0.52^A^	11.53±0.32^D^	13.61±0.32^D^	9.60±0.26^D^
2^nd^ week	Serum	25.60±0.27^A^	33.01±0.34^A^	29.86±0.27^A^	22.07±0.38^B^
	Gills	9.40±0.07^C^	14.19±0.34^C^	11.15±0.14^D^	8.53±0.22^D^
	Liver	26.62±0.41^A^	32.79±0.65^A^	23.84±0.34^B^	29.92±0.31^A^
	Kidney	17.50±0.38^B^	23.76±0.28^B^	21.09±0.27^B^	16.76±0.23^C^
	Musculature	10.47±0.10^C^	23.87±0.89^B^	15.93±0.39^D^	8.77±0.10^D^
3^rd^ week	Serum	25.40±0.45^A^	35.21±0.54^A^	29.86±0.27^A^	34.00±0.13^B^
	Gills	8.84±0.14^C^	18.19±0.24^C^	11.15±0.14^D^	8.91±0.19^D^
	Liver	26.09±0.42^A^	36.79±0.85^A^	23.84±0.34^B^	30.13±0.18^B^
	Kidney	16.93±0.49^B^	28.76±0.18^B^	21.09±0.27^B^	18.07±0.16^C^
	Musculature	10.68±0.28^C^	20.17±0.80^B^	15.93±0.39^D^	9.58±0.18^D^

Means within the same column of different letters are significantly different at p<0.05. *O. niloticus=Oreochromis niloticus,* IPM=Intraperitoneal injection of MC-LR, IMM=Immersion bath in MC-LR, FC=Feeding toxic cyanobacterial cells in pelleted diets, SE=Standard error, MC=Microcystins, MDA=Malondialdehyde

**Table-4:** Effects of cyanobacterial cells containing MC-LR on GR level (nmol/g wet tissue) in the serum, gills, liver, kidney, and muscular tissue of *O. niloticus* by different exposure routes.

Exposure period	Sample	Mean±SE			

		CTR	IPM	IMM	FC
1^st^ week	Serum	6.44±0.11^B^	5.79±0.08^B^	5.73±0.06^B^	5.43±0.12^D^
	Gills	2.50±0.09^E^	2.76±0.07^E^	4.31±0.12^C^	4.59±0.15^E^
	Liver	8.35±0.11^A^	7.31±0.04^A^	7.40±0.05^A^	8.48±0.17^A^
	Kidney	5.32±0.11^C^	5.63±0.06^B^	5.89±0.06^B^	6.55±0.09^C^
	Musculature	4.53±0.23^D^	4.12±0.13^C^	2.83±0.06^E^	2.59±0.08^G^
2^nd^ week	Serum	6.50±0.11^B^	5.52±0.09^B^	5.59±0.10^B^	6.66±0.06^C^
	Gills	4.46±0.18^D^	3.84±0.07^D^	3.92±0.08^D^	4.73±0.24^E^
	Liver	8.59±0.17^A^	7.13±0.05^A^	7.29±0.06^A^	8.93±0.18^A^
	Kidney	5.44±0.09^C^	5.50±0.09^B^	5.58±0.08^B^	5.64±0.06^D^
	Musculature	2.46±0.07^E^	2.70±0.06^E^	2.80±0.08^E^	2.86±0.07^G^
3^rd^ week	Serum	6.60±0.11^BC^	6.81±0.18^B^	6.90±0.41^B^	7.40±0.31^B^
	Gills	4.55±0.10^D^	4.75±0.30^D^	5.05±0.20^D^	5.65±0.15^D^
	Liver	8.35±0.06^A^	8.40±0.16^A^	8.67±0.21^A^	8.81±0.31^A^
	Kidney	5.41±0.06^C^	5.71±0.26^C^	5.80±0.09^C^	6.01±0.08^C^
	Musculature	2.62±0.10^E^	2.82±0.15^E^	3.62±0.14^E^	2.92±0.20^E^

Means within the same column of different letters are significantly different at p<0.05. *O. niloticus=Oreochromis niloticus*, IPM=Intraperitoneal injection of MCLR, IMM=Immersion bath in MCLR, FC=Feeding toxic cyanobacterial cells in pelleted diets, SE=Standard error, MC=Microcystins, GR=Glutathione reductase

**Table-5:** Effects of cyanobacterial cells containing MC-LR on GPx activities (U/g tissue) in the serum, gills, liver, kidney, and muscular tissue of *O. niloticus* by different exposure routes.

Exposure period	Sample	Mean±SE			

		CTR	IPM	IMM	FC
1^st^ Week	Serum	15.66±0.26^D^	13.93±0.34^D^	15.16±0.20^C^	16.29±0.33^D^
	Gills	17.76±0.23^C^	15.99±0.38^D^	17.10±0.48^C^	16.85±0.47^D^
	Liver	28.62±0.54^A^	26.36±0.68^A^	28.00±0.42^A^	33.25±0.98^A^
	Kidney	23.70±0.67^B^	20.68±0.32^C^	22.40±0.14^B^	25.81±0.36^B^
	Musculature	15.70±0.62^D^	14.06±0.75^D^	15.00±0.68^C^	17.14±0.17^C^
2^nd^ Week	Serum	24.19±0.34^B^	16.83±0.36^D^	20.73±0.62^B^	29.62±0.71^D^
	Gills	15.04±0.39^D^	12.06±0.20^E^	13.94±0.13^D^	19.30±0.18^G^
	Liver	28.37±0.52^A^	22.59±0.75^B^	26.30±0.26^A^	39.47±0.44^B^
	Kidney	18.18±0.33^C^	13.86±0.27^D^	15.03±0.15^C^	20.22±0.29^F^
	Musculature	15.68±0.90^D^	12.81±0.20^E^	13.99±0.70^D^	19.43±0.39^G^
3^rd^ Week	Serum	23.78±0.43^B^	30.21±0.24^C^	27.71±0.14^B^	33.20±0.64^C^
	Gills	15.41±0.50^D^	20.19±0.82^F^	19.12±0.62^C^	21.39±0.42^F^
	Liver	28.51±0.50^A^	46.27±1.13^A^	40.17±0.23^A^	48.57±1.53^A^
	Kidney	17.26±0.54^C^	22.05±0.29^E^	17.15±0.29^D^	25.00±0.89^E^
	Musculature	15.51±0.47^D^	20.24±0.50^E^	18.24±0.40^D^	24.14±0.40^E^

Means within the same column of different letters are significantly different at p<0.05. GPx=Glutathione peroxidase, *O. niloticus=Oreochromis niloticus*, IPM=Intraperitoneal injection of MC-LR, IMM=Immersion bath in MC-LR, FC=Feeding toxic cyanobacterial cells in pelleted diets, SE=Standard error, MC=Microcystins

**Table-6:** Effects of cyanobacterial cells containing MC-LR on CAT activity (μmol O^2^/min/mg protein/g wet tissue) in the serum, gills, liver, kidney, and muscular tissue of *O. niloticus* by different exposure routes.

Exposure period	Sample	Mean±SE			

		CTR	IPM	IMM	FC
1^st^ week	Serum	20.06±0.24^B^	19.06±0.33^C^	19.31±0.28^B^	22.88±0.40^F^
	Gills	8.29±0.24^C^	8.12±0.50^D^	8.37±0.17^C^	8.78±0.17^I^
	Liver	38.89±0.67^A^	37.28±0.39^A^	38.07±0.23^A^	44.71±0.62^B^
	Kidney	20.09±0.27^B^	18.63±0.33^C^	18.99±0.21^B^	21.33±0.59^F^
	Musculature	2.67±0.08^D^	2.26±0.05^E^	2.48±0.10^D^	2.86±0.05^IJ^
2^nd^ week	Serum	18.80±0.35^B^	16.95±0.37^C^	17.53±0.11^B^	29.00±0.33^D^
	Gills	7.76±0.27^C^	7.53±0.17^D^	7.99±0.08^C^	11.29±0.26^H^
	Liver	37.70±0.54^A^	33.00±0.26^B^	35.69±0.37^A^	46.76±0.38^B^
	Kidney	18.65±0.53^B^	16.70±0.37^C^	17.66±0.45^B^	24.34±0.32^E^
	Musculature	2.48±0.07^D^	2.08±0.03^E^	2.28±0.05^D^	3.12±0.08^JI^
3^rd^ week	Serum	18.84±0.76^B^	32.37±0.17^C^	23.67±0.27^C^	33.77±0.07^C^
	Gills	8.14±0.35^C^	15.14±0.20^E^	14.04±0.40^E^	16.08±0.48^E^
	Liver	37.91±0.53^A^	47.23±0.28^A^	45.33±0.28^A^	49.93±0.26^A^
	Kidney	18.09±0.71^B^	28.30±0.39^B^	22.58±0.17^B^	29.50±0.37^B^
	Musculature	12.49±0.15^D^	25.39±0.40^D^	21.72±0.13^D^	24.37±0.20^D^

Means within the same column of different letters are significantly different at p<0.05. CAT=Catalase. *O. niloticus=Oreochromis niloticus,* IPM=Intraperitoneal injection of MC-LR, IMM=Immersion bath in MC-LR, FC=Feeding toxic cyanobacterial cells in pelleted diets, SE=Standard error, MC=Microcystins

The activity of these biomarkers was significantly induced after 14 days of exposure in the liver, kidney, and serum (p<0.05) in exposed fish in IPM and FC groups and showed no significant changes in gills and musculature. The induction was evident mostly in the liver (which was the most affected organ).

## Discussion

Among the toxic algal blooms, cyanotoxins are of environmental and associated with health problems of the exposed fish [6], which occurs, in Egypt, annually during the warm period of the year [34].

This study was particularly focused on the hazardous impacts of the cyanobacterial algae, *M. aeruginosa* containing MC-LR not only on the clinicopathological changes of exposed fish but also to serum biochemical parameters of Nile tilapia, exposed through three different exposure methods for 3-week period.

Several reviews studied their effects on tissues of fish species using the single route of exposure, herein, the study focused principally on exposing the fish to three methods (oral, immersion, and injection routes), to study all ways, by which the toxins may gain access to fish. The toxicological effects of MCs have been demonstrated in fish, when they were exposed by oral route [18,35], by immersion [36], or IP injected [15,37].

The clinical examination during fish exposure revealed no mortalities were recorded in any of the three exposed groups and the control ones, although obvious behavioral, clinical, and PM changes were recorded and were directly in relation to the concentration and duration of the exposure to the cyanobacteria containing MC-LR [17,23].

The PM lesions noticed on examination of the internal organs including severely congested gills, swollen congested liver with hemorrhagic patches in tips, with congested engorged gall bladder of the exposed fish were like that observed in the toxicity of MCs in *C. carpio* L. [38], *Oncorhynchus mykiss* [39], and tilapias [37].

The toxicological impacts of MCs were clearly observed in various tissues of the exposed fish species; renal tissue (causing nephrotoxicity) of rainbow trout [39], as well as in the tilapia [40], in the branchial tissue of common carp [38], in the gastrointestinal tract of tilapia [40], in the heart of brown trout [41,42], and in the brain tissue [36].

In this study, regarding the histopathological findings of the exposed *O. niloticus*, moderate-to-severe histopathological changes were present in the livers, musculature, and gills of the exposed fish in the three experiments. Our findings were nearly in concordance to that of Fischer and Dietrich [14], Carbis *et al*. [43] in *C. carpio*, in *O. mossambicus* [15], and in *O. niloticus* [16].

The histopathological findings (for intraperitoneal injection, immersion, and oral route groups) of the exposed *O. niloticus* indicated that cyanobacteria produce potent toxins (Cyanotoxins, MCs) (which are mainly, hepatotoxins), which, not only be accumulated in the tissues of Nile tilapia [44], especially the liver [45] but also can change the architecture of the hepatocytes and impair their functions [46-48].

The histopathological findings of the liver of exposed fish showed vacuolization of hepatocytes [40,49], which may indicate an imbalance between the synthesis rate in the parenchymal cells and the release rate of these substances into the systemic circulation. Furthermore, necrotic hepatocytes found is evident previously described by Fischer and Dietrich [14], Mitsoura *et al*. [50] in *C. carpio*, which is caused by the hepatotoxic effects of MC-LR.

Our findings were mainly focused in the liver because the liver is the principle organ of detoxification of toxicants [51] and the accumulation of MCs in its tissue [52].

Regarding the effects on liver function enzymes, our finding showed significant elevation of serum ALT and serum AST enzymes with dose- and time-dependent manner. Our results were parallel to that of Rabergh *et al*. [38], Kopp and Heteša [53], who investigated their elevation in the serum of the MC intoxicated common carp after 2 h of intraperitoneal injection of toxin. In addition, our results were in concordance to that retrieved by Vajcova *et al*. [54], where their levels were similarly elevated in silver carp following intraperitoneal exposure to pure MC.

MC-LR-induced hepatotoxicity was noticed through the elevation of serum ALT and serum AST enzyme activities, which may be correlated to the increase in serum bile acid and the concentration of bilirubin [41,43].

Concerning the results of oxidative stress biomarkers, our findings showed elevated MDA levels and the activities of GR, GPx, and CAT enzymes, in the serum, kidney, and liver of the exposed fish among the 3 weeks of exposure to the control group. Furthermore, in a time-dependent manner, their activities were significantly induced after 14 days of exposure in liver, kidney, and serum in fish in IP injected and oral route exposed groups.

LPO is principally important for aquatic biota because they normally contain large quantities of highly unsaturated fatty acids [55].

These results were not parallel to that of Cazenave *et al*. [36], who reviewed significant changes in GR and GPx enzyme activities in gills of *Corydoras paleatus* exposed to MC-RR by immersion. The difference in the results may be due to species differences, different exposure period, and due to exposure to the different type of MC (MC-RR).

Furthermore, our findings were in concordance to that of experimentally exposed Nile tilapia, whereas, these biomarkers were elevated when tilapia orally exposed to cyanobacterial cells for 2-3 weeks [18], and IP injected with an acute dose of pure MCs [37].

## Conclusion

From our results, this study addresses the possible toxicological impacts of toxic *M. aeruginosa* containing MC-LR to Nile tilapia, and the results investigated that MC-LR is toxic to Nile tilapia in different routes of exposure as well as different doses.

## Authors’ Contributions

AMAK has carried out the research work and did data analysis. While HMRAL has designed, supervised the experiment, write and follow-up the manuscript. All authors read and approved the final manuscript.
